# Identification of positive cofactor 4 as a diagnostic and prognostic biomarker associated with immune infiltration in hepatocellular carcinoma

**DOI:** 10.1016/j.iliver.2023.08.007

**Published:** 2023-09-15

**Authors:** Liangliang Bai, Guan Liu, Gang Dou, Xiaojun He, Chenyu Gong, Hongbin Zhang, Kai Tan, Xilin Du

**Affiliations:** a School of Medicine, Yan'an University, Yan'an 716000, China; b Department of General Surgery, The Second Affiliated Hospital of Air Force Medical University, Xi'an 710038, China; c Xi'an Medical University, Xi'an 710068, China

**Keywords:** Hepatocellular carcinoma, Human positive cofactor 4, Prognosis, Risk signature

## Abstract

**Background and aims:**

Human positive cofactor 4 (PC4) is associated with the development and therapeutic resistance of several malignancies. However, the role of PC4 in hepatocellular carcinoma (HCC) remains obscure.

**Methods:**

The expression status of PC4 was explored in Gene Expression Omnibus and The Cancer Genome Atlas datasets. Subsequently, the prognostic and diagnostic significance of PC4 in HCC patients was analyzed. Functional enrichment analyses were conducted to explore biological functions and potential mechanisms. The CIBERSORT algorithm was used for immune infiltration analysis. The risk signature was constructed by LASSO-Cox regression and was validated with the International Cancer Genome Consortium dataset. Quantitative real-time polymerase chain reaction was used to verify the expression levels of all genes. Tumor Immune Dysfunction and Exclusion analysis evaluated immunotherapy response. Finally, using online databases, PC4-related competing endogenous RNA networks were constructed.

**Results:**

PC4 levels were significantly upregulated in HCC and positively correlated with the pathological grade and clinical stage. The PC4-high expression group showed worse prognosis. In addition, PC4 could distinguish between tumor and normal tissues with an area under the curve of 0.965. The PC4 level was associated with immune checkpoints and immune cell infiltration. In the training and validation sets, the eight-gene risk signature strongly correlated with HCC patient prognosis. Tumor Immune Dysfunction and Exclusion analysis showed that patients in both the PC4-low and low-risk groups were more likely to benefit from immunotherapy. Finally, an lncRNA/microRNA-101-3p/PC4 network was constructed.

**Conclusion:**

We confirmed PC4 as a diagnostic and prognostic biomarker in HCC patients. We also developed and validated an eight-gene risk signature, which will help in clinical decision-making. The competing endogenous RNA network could help explore the regulatory mechanisms of PC4 in HCC.

## 1 Introduction

Primary liver cancer (PLC) is a common malignancy with high annual incidence and mortality. According to the latest cancer statistics, PLC accounted for 900,000 new cases and 830,000 deaths globally in 2020, posing a severe threat to human life and health [1]. Hepatocellular carcinoma (HCC), the most common histopathological type of PLC, is highly invasive and insidious, leading to a high number of patients with advanced-stage disease at diagnosis who miss the optimal timing for radical surgical treatment [2]. In addition, patients who undergo radical surgical treatment have a recurrence of nearly 70% during the first five postoperative years [3]. The past few years have witnessed unprecedented advances in research on therapeutic drugs, targeted therapy, and immunotherapy, which have improved patient prognosis to some extent. However, significant heterogeneity surrounds drug efficacy and resistance, emphasizing the need for further research [4,5]. Therefore, finding effective biomarkers and potential therapeutic targets is urgent to help diagnose HCC and improve patient prognosis.

Human positive cofactor 4 (human PC4, yeast SUB1), a nuclear protein highly conserved during evolution, was extracted by purification from the human upstream stimulatory activity fraction in 1994 [6]. Earlier studies had shown that PC4 participates in the initiation, elongation, and termination of RNA polymerase II–mediated transcription [7]. However, extensive research on the PC4 gene has determined that PC4 is also closely associated with DNA damage repair, chromatin formation, and cell cycle regulation [[8], [9], [10], [11]]. Given that PC4 can interact with the P53 gene and form a positive feedback loop [12], it has long been thought to be a tumor-suppressor gene. However, the tumor-promoting effect of PC4 has recently received much attention from scholars. A strong correlation has been revealed between PC4 and the proliferation, metastasis, and therapy resistance of malignant tumors such as esophageal squamous cell carcinoma, non–small cell lung cancer, pancreatic ductal adenocarcinoma, breast cancer, and prostate cancer [[13], [14], [15], [16], [17]]. However, the role of PC4 in HCC is still unknown.

This study explored the PC4 gene level in HCC and the association between gene expression and diagnosis, prognosis, and immune infiltration. Additionally, this study constructed and validated a risk signature for effective assessment of patient prognosis and immunotherapy response. Finally, establishment of the competing endogenous RNA (ceRNA) network facilitates understanding of the regulatory mechanism of PC4 in HCC.

## 2 Materials and methods

### 2.1 Data acquisition and preparation

The expression and clinical data of 374 HCC and 50 normal samples were acquired as the primary analysis dataset from The Cancer Genome Atlas (TCGA) (https://portal.gdc.cancer.gov/), and the GSE25097, GSE36376, and GSE39791 datasets derived from Gene Expression Omnibus (GEO) (https://www.ncbi.nlm.nih.gov/geo/) were applied to identify differentially expressed genes between HCC and normal tissue. Expression and clinical data of 260 HCC samples were obtained from the International Cancer Genome Consortium (ICGC) dataset (https://dcc.icgc.org/) to validate the risk signature. The Xena database (https://xenabrowser.net/datapages/) was used to obtain data on HCC patient progression-free survival (PFS) and disease-specific survival (DSS).

### 2.2 Differential expression analysis

The Tumor Immune Estimation Resource (TIMER) database (http://timer.cistrome.org/) was used to analyze PC4 gene expression in 33 malignant tumors and normal tissues. The “limma” package was adopted to analyze the differential expression of the PC4 gene between HCC and normal tissues from TCGA and four GEO datasets [18]. The association between gene expression and different clinicopathological features of HCC patients was also analyzed by the “limma” package.

### 2.3 Diagnostic and prognostic analysis

The “pROC” package was used to plot receiver operating characteristic (ROC) curves for assessing the ability of the PC4 gene to distinguish Stage I–IV tumor tissues from normal tissues [19]. Time-dependent ROC curves for predicting HCC patient overall survival (OS) were plotted by the “timeROC” package [20]. Independent prognostic factors in HCC patients were explored using univariate and multivariate analyses. Differences in survival were shown by Kaplan–Meier (K–M) curves.

### 2.4 Identification of differentially expressed genes

The differentially expressed genes (DEGs) of the PC4-high and PC4-low groups were identified with the “limma” package. The filter criteria were *p*-value being <0.05 and log_2_FC value being >1/<−1. Heatmaps, and volcano plots were used to visualize the results.

### 2.5 Enrichment analyses

Gene Ontology (GO) and Kyoto Encyclopedia of Genes and Genomes (KEGG) enrichment analyses of DEGs were conducted to explore biological functions and potential mechanisms [21]. Gene set enrichment analysis (GSEA) was conducted to find PC4-associated signaling pathways [22].

### 2.6 Immune infiltration analysis

The “CIBERSORT” package was used to assess the level of immune infiltration of 22 immune cells in each HCC sample [23]. A box plot displayed the differences in immune cell infiltration between the PC4-high and PC4-low groups. The differences in the expression of 24 human leukocyte antigen (HLA) genes and eight immune checkpoint genes between the two groups were visualized in a box plot.

### 2.7 Construction and verification of risk signature

Genes coexpressed with PC4 were screened using the following criteria: a Pearson correlation coefficient >0.6/< −0.6 and a *p*-value <0.001. Differential expression analysis and univariate Cox analysis were conducted for screening for genes with high expression in HCC and associated with patient prognosis. The “glmnet” package was used to construct the risk signature through LASSO-Cox regression [24]. The ICGC dataset was used to validate the risk signature.

### 2.8 Prediction of immunotherapy response and drug sensitivity analysis

Based on the Tumor Immune Dysfunction and Exclusion (TIDE) database (http://tide.dfci.harvard.edu/), the patient response to immunotherapy was predicted using two common mechanisms of immune escape: T-cell dysfunction and exclusion. A larger TIDE score indicated a higher probability of immune escape and a worse response to immunotherapy in patients [25]. Drug sensitivity data were derived from Genomics of Drug Sensitivity in Cancer (https://www.cancerrxgene.org/). The “oncoPredict” package was used to analyze these data [26]. Box plots were used to visualize the results. The filter criterion was a *p*-value <0.001.

### 2.9 Construction of ceRNA network

With the ENCORI database (https://starbase.sysu.edu.cn/), the potential regulatory associations between microRNA (miRNA)–messenger RNA (mRNA) and long noncoding RNA (lncRNA)–miRNA were predicted [27]. The results were screened based on a correlation coefficient <−0.3 and a *p*-value <0.001. The miRNA–mRNA and lncRNA–miRNA–mRNA networks were visualized with the Cytoscape software.

### 2.10 Quantitative real-time polymerase chain reaction analysis

Five cell lines (L02, BEL7402, HCCLM3, HepG2, and Huh7) were purchased from Procell Life Science & Technology (Wuhan, China) and cultured according to the manufacturer's instructions. Six pairs of fresh specimens were collected from the Second Affiliated Hospital of Air Force Military Medical University. Quantitative real-time polymerase chain reaction was used to verify the expression levels of all genes. The primer sequence information is listed in Table S1.

### 2.11 Statistical analysis

In this study, all data analyses were conducted using R software (version 4.1.3). Differences between groups were compared using the Wilcoxon test. K–M analysis and log-rank tests were adopted for intergroup survival comparison. Independent prognostic factors were screened by univariate and multivariate analyses. A *p*-value of <0.05 was considered statistically significant.

## 3 Results

### 3.1 High PC4 expression in tumor tissues

**Fig. 1:**
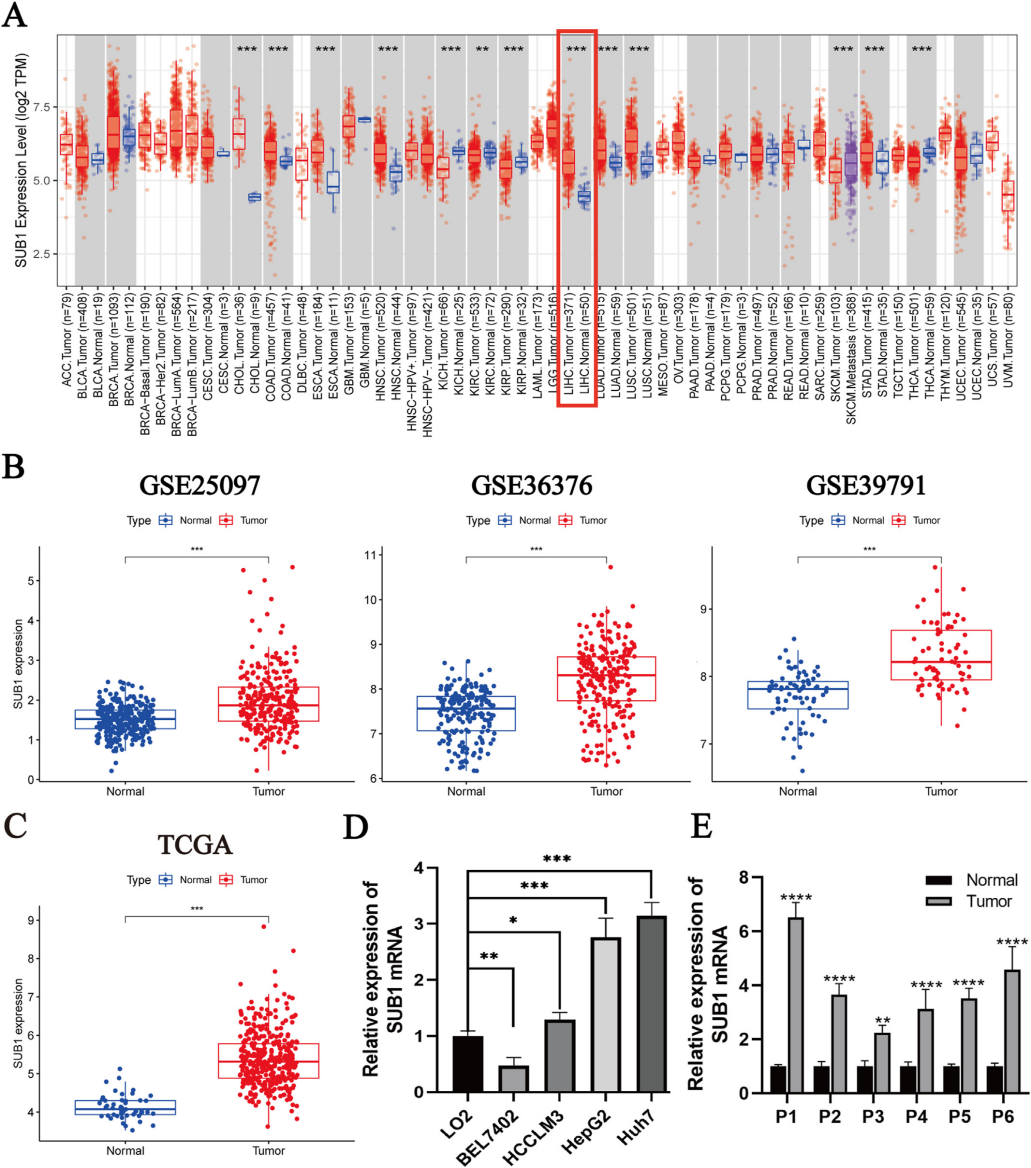
Expression status of positive cofactor 4 (PC4) in cancer. (A) Observation of PC4 expression level in pan-cancer from the Tumor Immune Estimation Resource database. (B) PC4 expression in hepatocellular carcinoma (HCC) and normal tissues from three Gene Expression Omnibus datasets (GSE25097, GSE36376, and GSE39791). (C) PC4 expression in HCC (*n* = 374) and adjacent normal liver tissues (*n* = 50) from The Cancer Genome Atlas database. (D) PC4 expression in five cell lines (LO2, BEL7402, HCCLM3, HepG2, and Huh7). (E) PC4 expression in six pairs of HCC and adjacent normal liver tissues. ∗*p* < 0.05, ∗∗*p* < 0.01, ∗∗∗*p* < 0.001, ∗∗∗∗*p* < 0.0001.

Pan-cancer analysis of the Tumor Immune Estimation Resource database showed significant differences in PC4 expression between 13 malignant tumor tissues and the adjacent normal ones. PC4 was highly expressed in nine malignancies, including cholangiocarcinoma, esophageal cancer, colon cancer, and HCC, while it was expressed at low levels in kidney chromophobe, kidney renal clear cell carcinoma, kidney renal papillary cell carcinoma, and thyroid cancer (Fig. 1A). According to differential expression analysis from the GEO and TCGA datasets, PC4 gene expression was significantly higher in HCC than in adjacent normal liver tissue (Fig. 1B, C). In addition, quantitative real-time polymerase chain reaction showed that the expression levels of PC4 in HCCLM3, HepG2, and Huh7 were significantly higher than in LO2 (Fig. 1D). The same results were obtained for HCC tissue samples (Fig. 1E).

### 3.2 Association of PC4 expression with the clinicopathological features and prognosis of HCC patients

**Fig. 2:**
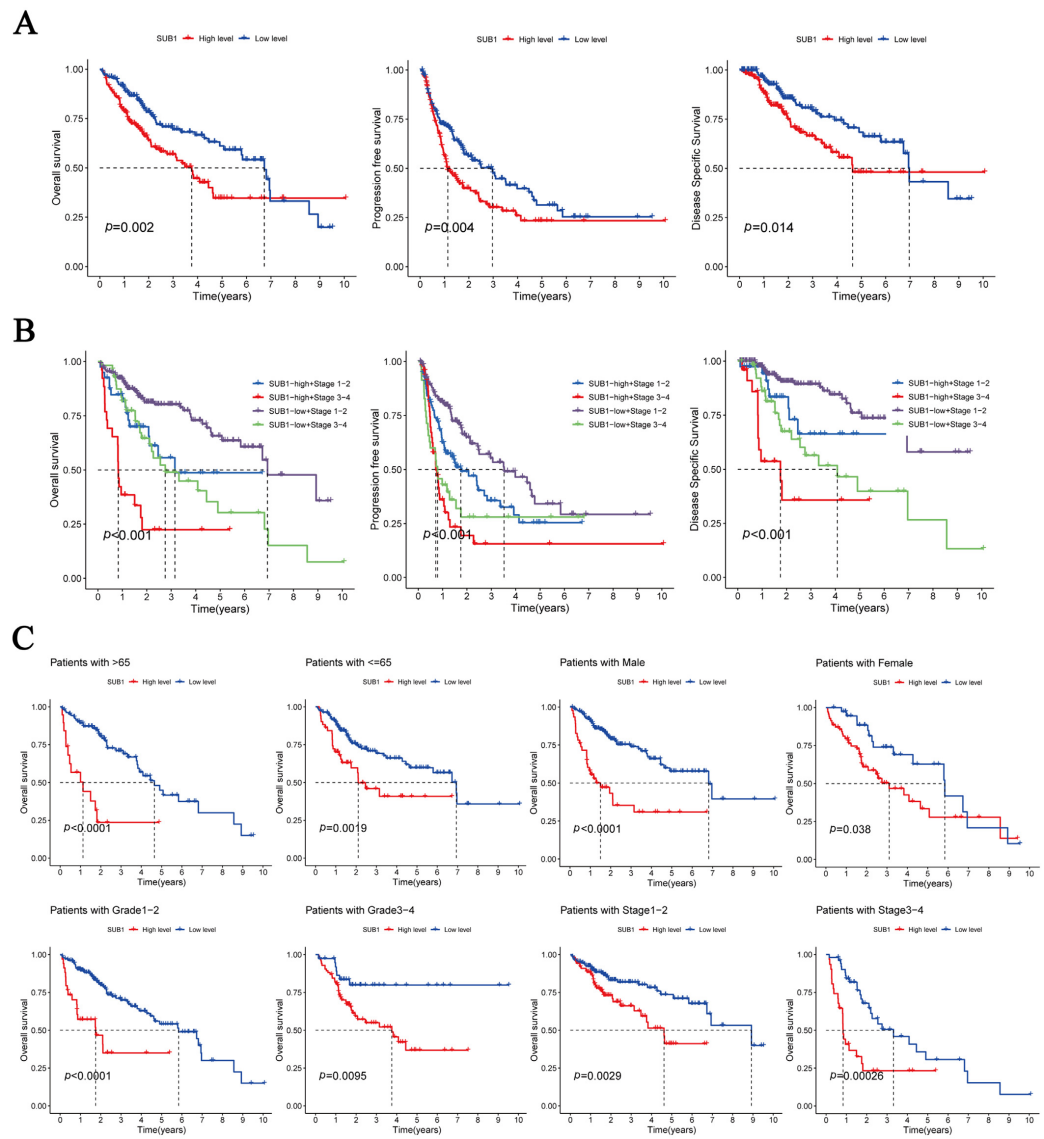
Correlation between positive cofactor 4 (PC4) expression level and survival and prognosis of hepatocellular carcinoma patients. (A) Kaplan–Meier survival curves for overall survival, progression-free survival, and disease-specific survival between the PC4-high and PC4-low groups. (B) Cumulative survival curves between the PC4-high and PC4-low groups under different clinical stages. (C) Kaplan–Meier survival curves for overall survival in different age, gender, grade, and stage subgroups.

Given the high PC4 gene expression in HCC, we further explored the association between PC4 expression and the clinicopathological features of HCC patients. The PC4 expression was not associated with the age or gender of HCC patients but was positively correlated with the pathological grade and clinical stage (Fig. S1). An association was also found between the expression of PC4 and the HCC patient prognosis. The PC4-high group had significantly lower OS, PFS, and DSS than the PC4-low group (Fig. 2A). The cumulative K–M curves for the clinical stages of combined HCC patients showed the same results. Patients in the PC4-high group at a late stage had the shortest survival time, whereas those in the PC4-low group at an early stage had the longest survival time (Fig. 2B). Subsequently, we assessed the influence of PC4 on HCC patient prognosis in different subgroups of age, gender, pathological grade, and clinical stage. The results implied a worse prognosis in the PC4-high group, irrespective of the subgroup (Fig. 2C).

### 3.3 Diagnostic and prognostic value of PC4

**Fig. 3:**
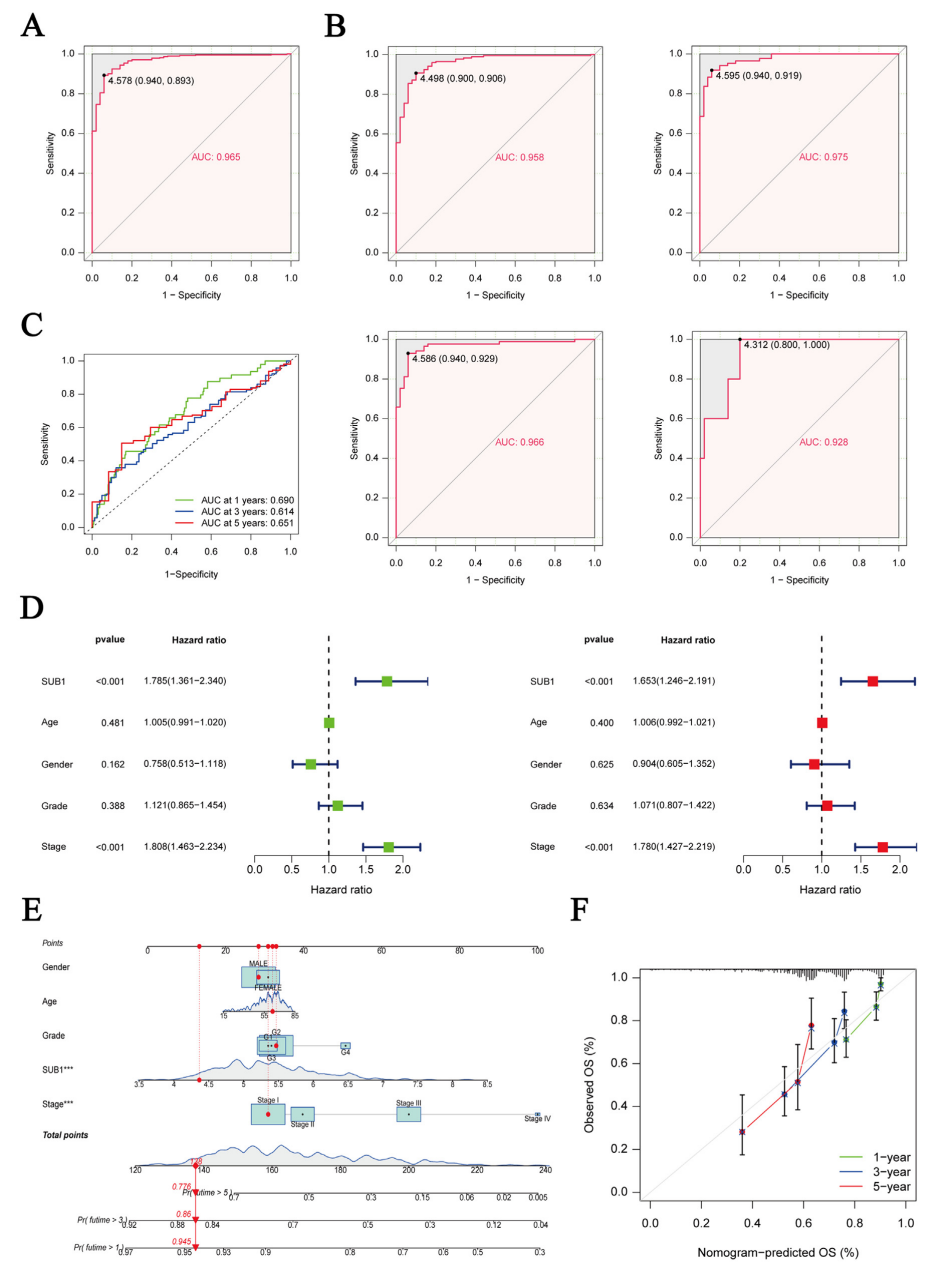
Diagnostic and prognostic value of positive cofactor 4 in hepatocellular carcinoma (HCC). (A) Receiver operating characteristic (ROC) curve for diagnosis to distinguish HCC from adjacent normal liver tissue. (B) ROC curve for diagnosis to distinguish Stage Ⅰ-Ⅳ HCC from adjacent normal liver tissue. (C) Time-dependent ROC curve to predict 1-, 3-, and 5-year survival rates of HCC patients. (D) Univariate and multivariate Cox analyses to identify independent prognostic factors. (E) Nomogram for predicting 1-, 3-, and 5-year survival rates of HCC patients by clinicopathological features and positive cofactor 4 expression level. (F) Calibration curve for evaluating the nomogram.

The area under the ROC curve (AUC) values were all larger than 0.9, suggesting that PC4 could distinguish Stage I-IV tumor tissues from normal tissues (Fig. 3A, B). In addition, PC4 exhibited good performance in predicting HCC patient prognosis. The 1-, 3-, and 5-year AUCs were 0.69, 0.61, and 0.65, respectively (Fig. 3C). According to univariate and multivariate analyses, PC4 expression was an independent prognostic factor for HCC patients (Fig. 3D). Subsequently, we further constructed a nomogram to predict the 1-, 3-, and 5-year survival of HCC patients, based on the clinicopathological features and PC4 expression level (Fig. 3E). The calibration curve showed that the nomogram had good predictive power (Fig. 3F).

### 3.4 Identification of DEGs and enrichment analysis

**Fig. 4:**
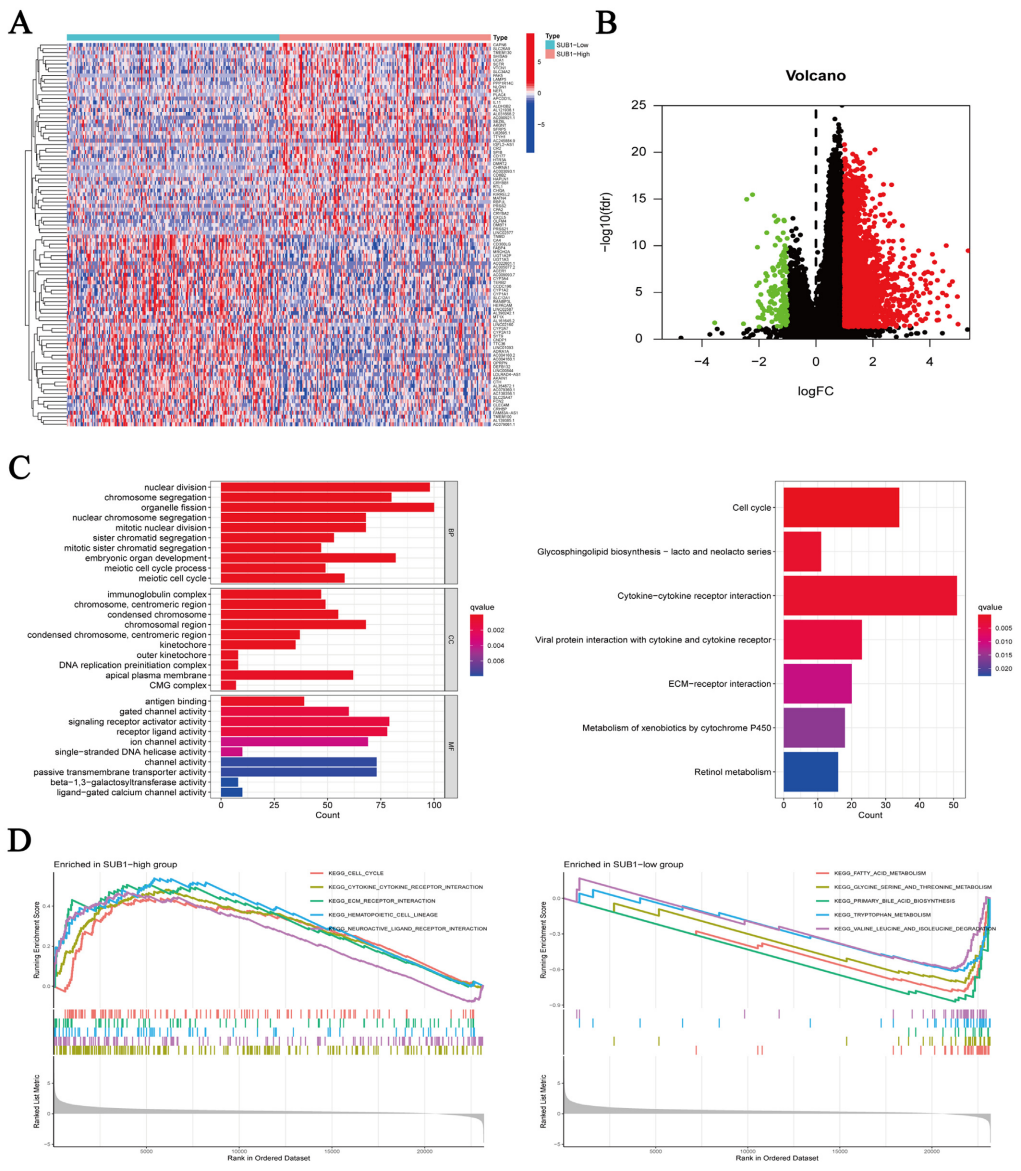
Identification of differential gene and enrichment analysis. (A) Heatmap showing 100 differentially expressed genes between the positive cofactor 4 (PC4)-high and PC4-low groups. (B) Volcano plot displaying the distribution of all differentially expressed genes based on log_2_FC > 1/< −1 and *p* < 0.05. (C) Gene Ontology and Kyoto Encyclopedia of Genes and Genomes enrichment analysis of all differentially expressed genes. (D) Gene set enrichment analysis for pathway enrichment between PC4-high and PC4-low groups.

We identified 2856 DEGs (2708 upregulated and 148 downregulated) in HCC (Table S2). A heatmap was generated showing the top 50 most significantly upregulated and downregulated genes in HCC (Fig. 4A). A volcano plot was generated to visualize the distribution of all DEGs (Fig. 4B). GO and KEGG analyses and GSEA were conducted to explore the potential mechanism of PC4 in HCC. GO enrichment analysis revealed that the DEGs were mainly enriched in biological processes including “nuclear division”, “chromosome segregation”, and “organelle fission”. KEGG enrichment analysis revealed that the DEGs were significantly enriched in the “cell cycle”, “glycosphingolipid biosynthesis-lacto and neolacto series”, and “cytokine–cytokine receptor interaction” pathways (Fig. 4C). According to the GSEA, the PC4-high group was primarily enriched in “cell cycle”, “cytokine–cytokine receptor interaction”, and “extracellular matrix (ECM) receptor interaction” signaling pathways, whereas the PC4-low group was enriched in “glycine, serine and threonine metabolism”, “fatty acid metabolism”, and “primary bile acid biosynthesis” (Fig. 4D).

### 3.5 Immune infiltration analysis between groups

The association between PC4 and tumor-infiltrating immune cells was further analyzed using the “CIBERSORT” algorithm. Memory B cells, activated dendritic cells, and M0 macrophages were differentially expressed between the PC4-high and PC4-low groups (Fig. S2A). Subsequently, correlation analysis between PC4 expression, 24 HLA genes, and eight immune checkpoint genes showed that all genes were overexpressed in the PC4-high group (Figs. S2C and D). The difference in TIDE scores indicated a higher incidence of immune escape and a lower response rate to immunotherapy in the PC4-high group (Fig. S2B).

### 3.6 Construction and validation of an eight-gene risk signature

**Fig. 5:**
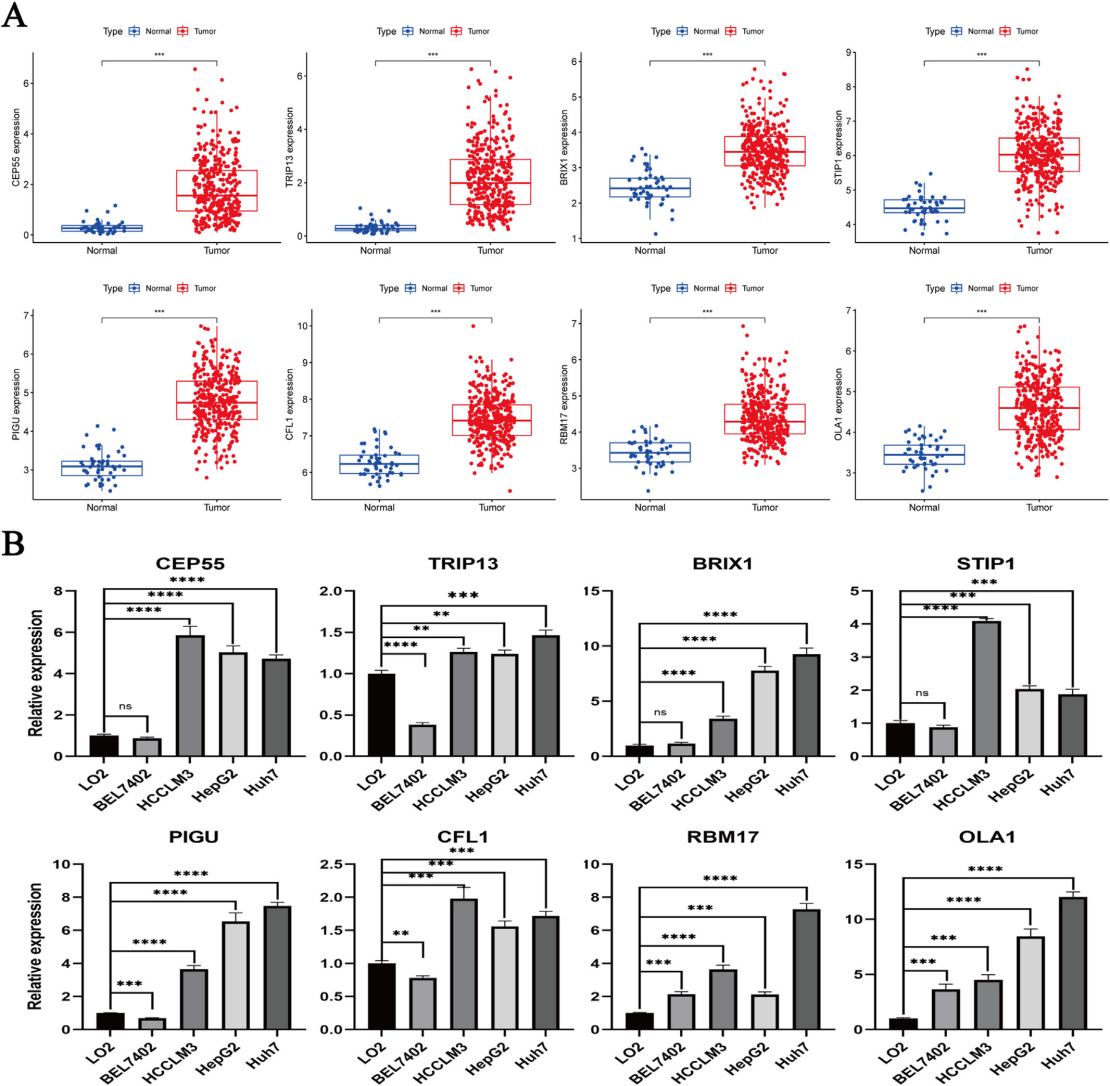
Expression levels of eight genes in the risk signature. (A) Expression levels of eight genes in hepatocellular carcinoma (*n* = 374) and adjacent normal liver tissues (*n* = 50) from The Cancer Genome Atlas database. (B) Expression levels of eight genes in five cell lines (LO2, BEL7402, HCCLM3, HepG2, and Huh7). ∗*p* < 0.05, ∗∗*p* < 0.01, ∗∗∗*p* < 0.001, ∗∗∗∗*p* < 0.0001.

**Fig. 6:**
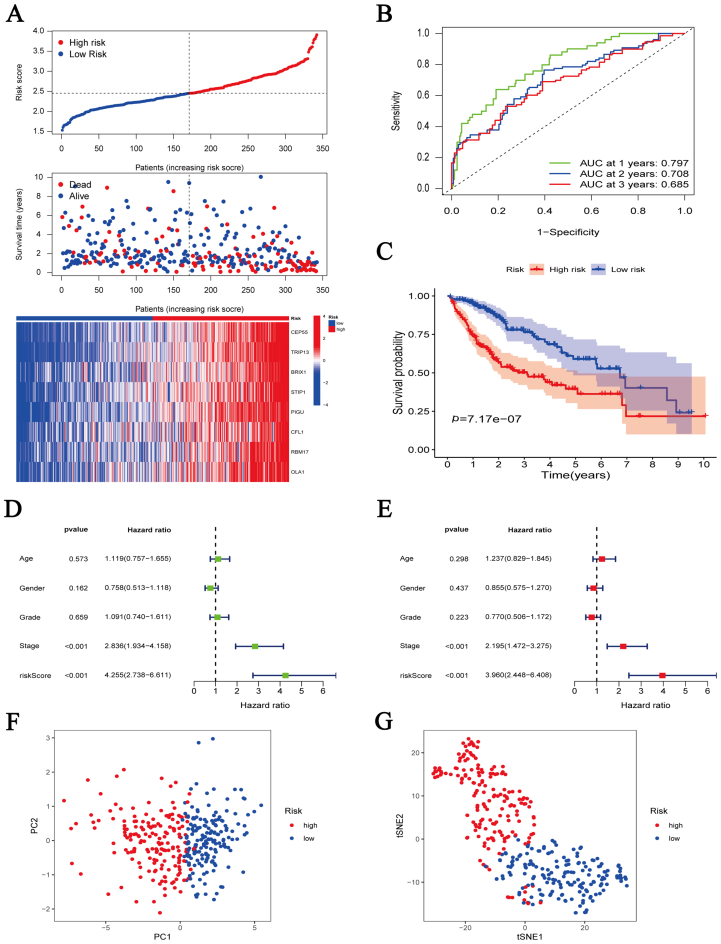
Survival analysis of the eight-gene risk signature in the training cohort (The Cancer Genome Atlas cohort). (A) Risk score distribution, survival status, and gene expression in high-risk and low-risk groups. (B) Time-dependent receiver operating characteristic curve to predict 1-, 2-, and 3-year survival rates of hepatocellular carcinoma patients. (C) Overall survival Kaplan–Meier curve between the high-risk and low-risk groups. (D, E) Univariate (D) and multivariate (E) Cox analysis to identify independent prognostic factors. (F, G) Principal component analysis (F) and t-distributed stochastic neighbor embedding analysis (G) for the two groups.

**Fig. 7:**
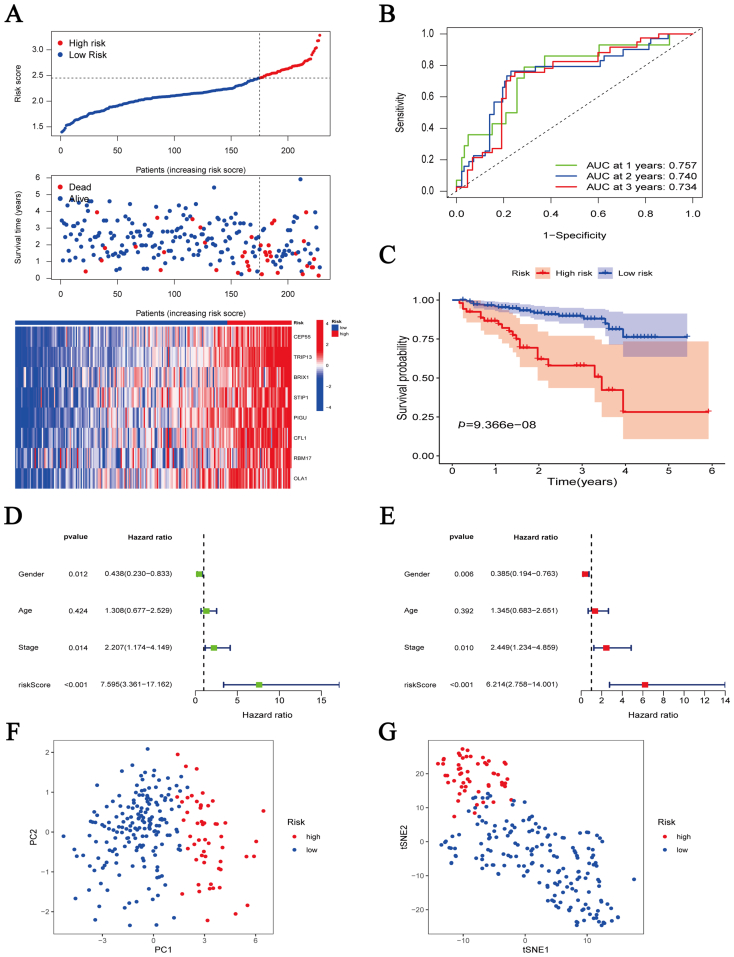
Survival analysis of the eight-gene risk signature in the test cohort (International Cancer Genome Consortium cohort). (A) Risk score distribution, survival status, and gene expression in the high-risk and low-risk groups. (B) Time-dependent receiver operating characteristic curve to predict 1-, 2-, and 3-year survival rates of hepatocellular carcinoma patients. (C) Overall survival Kaplan–Meier curve between the high-risk and low-risk groups. (D, E) Univariate (D) and multivariate (E) Cox analysis to identify independent prognostic factors. (F, G) Principal component analysis (F) and t-distributed stochastic neighbor embedding analysis (G) for the two groups.

To predict HCC patient prognosis, we constructed a risk signature by coexpression analysis. A heatmap showed that 92 PC4-related genes were overexpressed in HCC (Fig. S3A). The univariate Cox analysis further revealed that 77 genes were related to HCC patient prognosis (Fig. S3B). Subsequently, we constructed and validated an eight-gene risk signature (risk score = [0.074 ∗ CEP55] + [0.091 ∗ TRIP13] + [0.100 ∗ BRIX1] + [0.081 ∗ STIP1] + [0.191 ∗ PIGU] + [0.038 ∗ CFL1] + [0.017 ∗ RBM17] + [0.178 ∗ OLA1]) (Table S3). These eight genes are highly expressed in HCC samples from TCGA (Fig. 5A). Meanwhile, the same results were shown in cell lines. The expression of all genes in HCCLM3, HepG2, and Huh7 was significantly higher than in LO2 (Fig. 5B). The ROC curve in the training set confirmed the predictive power of this risk signature for HCC patient prognosis. The 1-, 2-, and 3-year AUC values were 0.79, 0.70, and 0.68, respectively (Fig. 6B). The number of deaths in HCC patients increased correspondingly with an increase in the risk score (Fig. 6A). The same results were obtained during K–M survival curve analysis. The high-risk group presented a significantly worse prognosis than the low-risk one (Fig. 6C). According to univariate and multivariate analysis, the risk score was an independent prognostic factor for HCC patients (Fig. 6D, E). Principal component analysis (PCA) and t-distributed stochastic neighbor embedding (t-SNE) analysis revealed that the risk signature could distinguish between high- and low-risk patients (Fig. 6F, G). The aforementioned results from the training set were confirmed in the validation set. The 1-, 2-, and 3-year AUC values of the validation set were 0.75, 0.74, and 0.73, respectively (Fig. 7A-G).

Additionally, the influence of the risk signature on HCC patient prognosis in different subgroups of age, gender, pathological grade, and clinical stage was further analyzed. The results revealed a worse prognosis in the high-risk group in any subgroup in the training set and validation set (Fig. S4). The clinical data of HCC patients from TCGA (training set) and the ICGC datasets (validation set) are provided in Table S4. Subsequently, we constructed a nomogram with the risk signature and clinicopathological features to predict the survival rates of HCC patients at 1, 2, and 3 years (Fig. S5A). The calibration curve revealed that the nomogram had good predictive power (Fig. S5B).

Finally, TIDE analysis assessed the correlation between the risk signature and immunotherapy response. Patients with high scores were unlikely to respond to immunotherapy, while the opposite was true for patients with low scores (Fig. S5C). Drug sensitivity analysis showed that patients in the high-risk group were sensitive to sorafenib, axitinib, cytarabine, entinostat, fludarabine, irinotecan, obatoclax mesylate, oxaliplatin, ribociclib, and selumetinib. Conversely, patients in the low-risk group were sensitive to 5-fluorouracil, alpelisib, cediranib, dasatinib, fulvestrant, lapatinib, osimertinib, pevonedistat, pictilisib, and taselisib (Fig. S6).

### 3.7 Construction of ceRNA network based on PC4

**Fig. 8:**
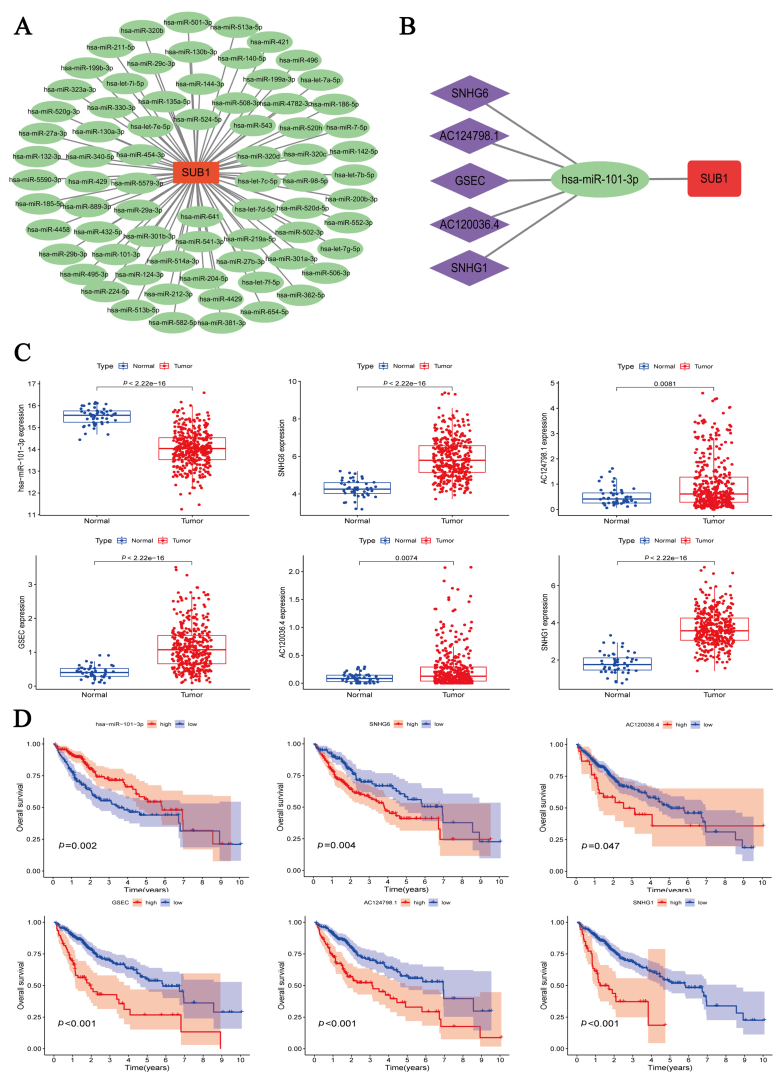
Construction of positive cofactor 4 (PC4)-associated competing endogenous RNA network. (A) microRNA (miRNA)–messenger RNA (mRNA) network composed of PC4 and 73 miRNAs. (B) long noncoding RNA (lncRNA)–miRNA–mRNA network composed of PC4, miRNA-101, and five lncRNAs. (C) Expression differences of miRNA-101 and five lncRNAs in hepatocellular carcinoma and adjacent normal liver tissues based on The Cancer Genome Atlas cohort. (D) Overall survival Kaplan–Meier curves of miRNA-101 and five lncRNAs based on The Cancer Genome Atlas cohort.

To explore the potential upstream regulators of PC4, we constructed a PC4-related ceRNA network. First, 73 possible upstream miRNAs were obtained from the ENCORI online database, and the miRNA–mRNA network was constructed through Cytoscape software (Fig. 8A). Correlation analysis between PC4 and miRNAs further identified miRNA-101-3p as a potential regulatory miRNA of PC4 (Fig. S7A). Subsequently, we obtained 49 possible lncRNAs to explore the upstream regulatory lncRNAs of miRNA-101-3p. Correlation analysis of miRNA-101-3p with lncRNAs ultimately identified five potential lncRNAs. The expression of these lncRNAs was positively correlated with PC4 and negatively with miRNA-101-3p (Fig. S7B–F, S8). Finally, the lncRNA/miRNA-101-3p/PC4 network was constructed (Fig. 8B). Boxplots and K–M curves were generated to visualize the differences in expression and OS of miRNA-101-3p and the five lncRNAs. miRNA-101-3p had low expression in HCC, and HCC patients with low expression showed a worse prognosis. In addition, all five lncRNAs were highly expressed in HCC and predicted a poorer prognosis (Fig. 8C, D).

## 4 Discussion

As the most common histopathological type of PLC, HCC is highly invasive and insidious, accounting for the difficulty clinicians face in diagnosing and treating HCC. Alpha-fetoprotein (AFP) is the most commonly used diagnostic and screening biomarker for HCC. However, its sensitivity and specificity still do not meet clinical needs [28]. In recent years, targeted therapy and immunotherapy for HCC have achieved remarkable efficacy, but the heterogeneity in efficacy and drug resistance remains to be addressed [4,5]. Thus, searching for effective biomarkers and potential therapeutic targets is urgent to offer new strategies for diagnosing and treating HCC. PC4 is a highly conserved nuclear protein implicated in transcriptional regulation, DNA damage repair, and chromatin formation [29]. Moreover, overexpression of PC4 is significantly associated with proliferation, metastasis, and treatment resistance in various malignancies. PC4 accelerates the development of pancreatic ductal adenocarcinoma by activating the mTOR/p70s6k signaling pathway [16]. PC4 can also bind directly to the c-Myc promoter and regulate its transcription, which induces the Warburg effect to promote breast cancer progression [30]. However, the role of PC4 in HCC remains largely understudied. This study comprehensively analyzed the diagnostic and prognostic value of PC4 in HCC and constructed a risk signature to effectively assess the prognosis of HCC patients. The construction of the ceRNA network facilitated the understanding of the regulatory mechanisms of PC4 in HCC.

PC4 is highly expressed in various malignancies, with the most significant differential expression in HCC. In addition, a positive correlation was found between the PC4 expression and the pathological grade and clinical stage of HCC patients, suggesting that PC4 potentially participates in the development of HCC. The present study analyzes the value of PC4 in the diagnosis and prognosis of HCC. The diagnostic ROC curve substantiated the ability of PC4 to distinguish tumor tissue from normal tissue. Survival analysis of OS, PFS, and DSS showed that high PC4 expression correlated with a poor HCC patient prognosis. Additionally, the prognostic ROC curve and nomogram indicated that PC4 could effectively predict the 1-, 3-, and 5-year survival rates of HCC patients. The aforementioned results imply that PC4 has huge potential as a diagnostic and prognostic biomarker for HCC.

To understand the role of PC4 in HCC, we identified DEGs and conducted functional enrichment analyses. The PC4-high group was mainly associated with the “cell cycle”, “cytokine–cytokine receptor interaction”, and “ECM receptor interaction” signaling pathways. This result is consistent with the functions of PC4 documented in the literature. Subsequently, we further explored the association between the expression level of PC4, immune infiltration, and the immunotherapy response. The PC4 expression positively correlated with numerous HLA genes and immune checkpoint–related genes. Additionally, patients with high PC4 expression had a higher probability of immune escape, a poorer response to immunotherapy, and shorter survival. This result is caused by the tumor-suppressive microenvironment induced by the overexpression of immune checkpoint genes [31,32].

We found that the ability of a single gene to assess HCC patient prognosis was limited. Therefore, to improve the predictive power, we constructed and validated an eight-gene risk signature (risk score = [0.074 ∗ CEP55] + [0.091 ∗ TRIP13] + [0.100 ∗ BRIX1] + [0.081 ∗ STIP1] + [0.191 ∗ PIGU] + [0.038 ∗ CFL1] + [0.017 ∗ RBM17] + [0.178 ∗ OLA1]). Most genes in this risk signature are strongly associated with the prognosis of HCC or other malignancies. CEP55 is located in the chromosomal region 10q23 and is important in cell division [33]. CEP55 promotes HCC invasion and migration through the JAK2/STAT3/MMPs signaling pathway [34]. As an AAA + ATPase that can promote the assembly or degradation of protein complexes, TRIP13 is significantly upregulated in various malignancies, including HCC [35]. Moreover, TRIP13 can interact with ACTN4 to induce HCC invasion and metastasis through the AKT/mTOR pathway [36]. A study that constructed a prognostic risk signature for HCC based on RNA-binding proteins showed an association between BRIX1 and poor prognosis of HCC patients [37]. STIP1, called heat shock protein–organizing protein, is reportedly overexpressed in HCC and accelerates cancer-cell growth and migration by interacting with Axin to activate β-catenin/TCF signaling [38]. In addition, it has been reported that STIP1 in serum can be used as an indicator of microvascular invasion and can be used to predict prognosis and response to trans arterial chemoembolisation treatment in HCC patients [39]. PIGU, also called cell division cycle 91-like 1 (CDC91L1), promotes HCC progression by activating the NF-κB pathway and promoting immune escape [40]. CFL1 is an important actin depolymerization factor family member, widely found in eukaryotes. Hypoxia can induce CFL1 expression and thus activate the PLD1/AKT pathway to promote HCC progression [41]. RBM17 is also known as splicing factor 45 (SPF45). Knockdown of RBM17 significantly inhibits cancer-cell proliferation and arrested cells in the G2/M phase [42]. Moreover, OLA1 is implicated in cellular processes such as protein translation, signal transduction, and cell proliferation [43]. OLA1 is significantly expressed in HCC, and knockdown of this gene suppresses the progression of cancer cells [44]. These studies overlap in their assertion that the risk score correlates with HCC patient prognosis. Moreover, various analyses have shown that this risk score is reliable in predicting the prognosis of HCC patients. TIDE analysis showed that patients with high scores were more likely to be unresponsive to immunotherapy. Drug sensitivity analysis screened the drugs to which the high-risk and low-risk groups are sensitive. These results will help guide clinicians in making treatment decisions.

To better understand the role of PC4 in HCC, we constructed a lncRNA/miRNA-101-3p/PC4 network. Most regulatory relationships in this network have been reported in malignancies. The lncRNA SNHG6 improves E2F8 expression by associating with miR-101-3p, thus promoting proliferation and angiogenesis in cholangiocarcinoma [45]. In HCC, SNHG6 upregulates ZEB1 expression by binding miR-101-3p and, in combination with downregulation of Smad7, induces epithelial–mesenchymal transition, speeding up cancer-cell metastasis [46]. An SNX16- and PAPOLG-based study showed that the GSEC/miR-101-3p/SNX16/PAPOLG axis is significantly associated with HCC patient prognosis [47]. Reportedly, the lncRNA SNHG1 interacts with miR-101-3p to promote tumor progression. The SNHG1/miR-101-3p/SOX9/Wnt/β-catenin axis promotes the progression of non–small cell lung cancer [48]. miR-101-3p is closely associated with programmed cell death, such as autophagy and apoptosis, playing an important role in HCC [49]. miR-101-3p can bind PYGB and inhibit its expression, while the overexpression of PYGB, in turn, inhibits the regulation of miR-101-3p on HCC cell invasion, proliferation, and migration [50]. However, the association between miR-101–3p and PC4 in HCC remains unknown. The construction of this lncRNA/miRNA-101-3p/PC4 network may bridge that knowledge gap.

In conclusion, this study demonstrates the potential of PC4 as a diagnostic and prognostic biomarker for HCC. Moreover, the PC4-based risk signature could guide clinicians in evaluating the prognosis and response to immunotherapy of HCC patients.

## Funding

This research did not receive any specific grant from funding agencies in the public, commercial, or not-for-profit sectors.

## Author contributions

LB, GL, and GD conceived the study. XH, CG, and HZ conducted data collection and collation. LB and GL were responsible for data analysis. LB wrote the manuscript, and KT and XD reviewed and revised it. All authors reviewed the manuscript. LB, GL, and GD contributed equally to this work.

## Acknowledgments

We are grateful to the The Cancer Genome Atlas, Gene Expression Omnibus, and International Cancer Genome Consortium databases for providing data for this study. We thank the “ENCORI” and “Tumor Immune Dysfunction and Exclusion” databases and “R” and “Cytoscape” softwares for their help in data analysis.

## Declaration of competing interest

The authors declare that the research was conducted in the absence of any commercial or financial relationships that could be construed as a potential competing interest.

## Data availability statement

In this study, the data for HCC were freely obtained from publicly accessible datasets: TCGA (https://portal.gdc.cancer.gov/), GEO (https://www.ncbi.nlm.nih.gov/geo/), and the ICGC database (https://dcc.icgc.org/).

## Ethics statement

Ethics approval was waived for this study because our data were retrieved from public databases.

## Informed consent

Informed consent was not applicable because our data were from public databases.

## References

[1] Sung H., Ferlay J., Siegel R.L. (2021). Global cancer statistics 2020: GLOBOCAN estimates of incidence and mortality worldwide for 36 cancers in 185 countries. CA Cancer J Clin.

[2] Park J.W., Chen M., Colombo M. (2015). Global patterns of hepatocellular carcinoma management from diagnosis to death: the BRIDGE Study. Liver Int.

[3] Roayaie S., Obeidat K., Sposito C. (2013). Resection of hepatocellular cancer ≤2 cm: results from two Western centers. Hepatology.

[4] Huang A., Yang X.R., Chung W.Y. (2020). Targeted therapy for hepatocellular carcinoma. Signal Transduct Targeted Ther.

[5] Cheng A.L., Hsu C., Chan S.L. (2020). Challenges of combination therapy with immune checkpoint inhibitors for hepatocellular carcinoma. J Hepatol.

[6] Ge H., Roeder R.G. (1994). Purification, cloning, and characterization of a human coactivator, PC4, that mediates transcriptional activation of class II genes. Cell.

[7] Calvo O., Manley J.L. (2005). The transcriptional coactivator PC4/Sub1 has multiple functions in RNA polymerase II transcription. EMBO J.

[8] Mortusewicz O., Roth W., Li N. (2008). Recruitment of RNA polymerase II cofactor PC4 to DNA damage sites. J Cell Biol.

[9] Mortusewicz O., Evers B., Helleday T. (2016). PC4 promotes genome stability and DNA repair through binding of ssDNA at DNA damage sites. Oncogene.

[10] Das C., Hizume K., Batta K. (2006). Transcriptional coactivator PC4, a chromatin-associated protein, induces chromatin condensation. Mol Cell Biol.

[11] Dhanasekaran K., Kumari S., Boopathi R. (2016). Multifunctional human transcriptional coactivator protein PC4 is a substrate of Aurora kinases and activates the Aurora enzymes. FEBS J.

[12] Mondal P., Saleem S., Sikder S. (2019). Multifunctional transcriptional coactivator PC4 is a global co-regulator of p53-dependent stress response and gene regulation. J Biochem.

[13] Peng Y., Yang J., Zhang E. (2012). Human positive coactivator 4 is a potential novel therapeutic target in non-small cell lung cancer. Cancer Gene Ther.

[14] Qian D., Zhang B., Zeng X.L. (2014). Inhibition of human positive cofactor 4 radiosensitizes human esophageal squmaous cell carcinoma cells by suppressing XLF-mediated nonhomologous end joining. Cell Death Dis.

[15] Wang Q., Ma L., Chen L. (2021). Knockdown of PC4 increases chemosensitivity of Oxaliplatin in triple negative breast cancer by suppressing mTOR pathway. Biochem Biophys Res Commun.

[16] Su X., Yang Y., Ma L. (2020). Human positive coactivator 4 affects the progression and prognosis of pancreatic ductal adenocarcinoma via the mTOR/P70s6k signaling pathway. OncoTargets Ther.

[17] Chakravarthi B.V., Goswami M.T., Pathi S.S. (2016). MicroRNA-101 regulated transcriptional modulator SUB1 plays a role in prostate cancer. Oncogene.

[18] Ritchie M.E., Phipson B., Wu D. (2015). Limma powers differential expression analyses for RNA-sequencing and microarray studies. Nucleic Acids Res.

[19] Robin X., Turck N., Hainard A. (2011). pROC: an open-source package for R and S+ to analyze and compare ROC curves. BMC Bioinf.

[20] Blanche P., Dartigues J.F., Jacqmin-Gadda H. (2013). Estimating and comparing time-dependent areas under receiver operating characteristic curves for censored event times with competing risks. Stat Med.

[21] Yu G., Wang L.G., Han Y. (2012). clusterProfiler: an R package for comparing biological themes among gene clusters. OMICS.

[22] Subramanian A., Tamayo P., Mootha V.K. (2005). Gene set enrichment analysis: a knowledge-based approach for interpreting genome-wide expression profiles. Proc Natl Acad Sci U S A.

[23] Chen B., Khodadoust M.S., Liu C.L. (2018). Profiling tumor infiltrating immune cells with CIBERSORT. Methods Mol Biol.

[24] Friedman J., Hastie T., Tibshirani R. (2010). Regularization paths for generalized linear models via coordinate descent. J Stat Software.

[25] Jiang P., Gu S., Pan D. (2018). Signatures of T cell dysfunction and exclusion predict cancer immunotherapy response. Nat Med.

[26] Maeser D., Gruener R.F., Huang R.S. (2021). oncoPredict: an R package for predicting in vivo or cancer patient drug response and biomarkers from cell line screening data. Briefings Bioinf.

[27] Li J.H., Liu S., Zhou H. (2014). starBase v2.0: decoding miRNA-ceRNA, miRNA-ncRNA and protein-RNA interaction networks from large-scale CLIP-Seq data. Nucleic Acids Res.

[28] Tateishi R., Yoshida H., Matsuyama Y. (2008). Diagnostic accuracy of tumor markers for hepatocellular carcinoma: a systematic review. Hepatol Int.

[29] Chen L., Liao F., Wu J. (2021). Acceleration of ageing via disturbing mTOR-regulated proteostasis by a new ageing-associated gene PC4. Aging Cell.

[30] Luo P., Zhang C., Liao F. (2019). Transcriptional positive cofactor 4 promotes breast cancer proliferation and metastasis through c-Myc mediated Warburg effect. Cell Commun Signal.

[31] Patsoukis N., Wang Q., Strauss L. (2020). Revisiting the PD-1 pathway. Sci Adv.

[32] Rowshanravan B., Halliday N., Sansom D.M. (2018). CTLA-4: a moving target in immunotherapy. Blood.

[33] Zhao W.M., Seki A., Fang G. (2006). Cep55, a microtubule-bundling protein, associates with centralspindlin to control the midbody integrity and cell abscission during cytokinesis. Mol Biol Cell.

[34] Li M., Gao J., Li D. (2018). CEP55 promotes cell motility via JAK2⁻STAT3⁻MMPs cascade in hepatocellular carcinoma. Cells.

[35] Ju L., Li X., Shao J. (2018). Upregulation of thyroid hormone receptor interactor 13 is associated with human hepatocellular carcinoma. Oncol Rep.

[36] Zhu M.X., Wei C.Y., Zhang P.F. (2019). Elevated TRIP13 drives the AKT/mTOR pathway to induce the progression of hepatocellular carcinoma via interacting with ACTN4. J Exp Clin Cancer Res.

[37] Wang L., Zhang Z., Li Y. (2020). Integrated bioinformatic analysis of RNA binding proteins in hepatocellular carcinoma. Aging (Albany NY).

[38] Luo X., Liu Y., Ma S. (2018). STIP1 is over-expressed in hepatocellular carcinoma and promotes the growth and migration of cancer cells. Gene.

[39] Ma X.L., Tang W.G., Yang M.J. (2020). Serum STIP1, a novel indicator for microvascular invasion, predicts outcomes and treatment response in hepatocellular carcinoma. Front Oncol.

[40] Wei X., Yang W., Zhang F. (2020). PIGU promotes hepatocellular carcinoma progression through activating NF-κB pathway and increasing immune escape. Life Sci.

[41] Yao B., Li Y., Chen T. (2021). Hypoxia-induced cofilin 1 promotes hepatocellular carcinoma progression by regulating the PLD1/AKT pathway. Clin Transl Med.

[42] Li C., Ge S., Zhou J. (2020). Exploration of the effects of the CYCLOPS gene RBM17 in hepatocellular carcinoma. PLoS One.

[43] Verstraeten N., Fauvart M., Versées W. (2011). The universally conserved prokaryotic GTPases. Microbiol Mol Biol Rev.

[44] Huang S., Zhang C., Sun C. (2020). Obg-like ATPase 1 (OLA1) overexpression predicts poor prognosis and promotes tumor progression by regulating P21/CDK2 in hepatocellular carcinoma. Aging (Albany NY).

[45] Wang H., Wang L., Tang L. (2020). Long noncoding RNA SNHG6 promotes proliferation and angiogenesis of cholangiocarcinoma cells through sponging miR-101-3p and activation of E2F8. J Cancer.

[46] Chang L., Yuan Y., Li C. (2016). Upregulation of SNHG6 regulates ZEB1 expression by competitively binding miR-101-3p and interacting with UPF1 in hepatocellular carcinoma. Cancer Lett.

[47] Hu S., Zhang J., Guo G. (2022). Comprehensive analysis of GSEC/miR-101-3p/SNX16/PAPOLG axis in hepatocellular carcinoma. PLoS One.

[48] Cui Y., Zhang F., Zhu C. (2017). Upregulated lncRNA SNHG1 contributes to progression of non-small cell lung cancer through inhibition of miR-101-3p and activation of Wnt/β-catenin signaling pathway. Oncotarget.

[49] Xu L., Beckebaum S., Iacob S. (2014). MicroRNA-101 inhibits human hepatocellular carcinoma progression through EZH2 downregulation and increased cytostatic drug sensitivity. J Hepatol.

[50] Cui G., Wang H., Liu W. (2020). Glycogen phosphorylase B is regulated by miR101-3p and promotes hepatocellular carcinoma tumorigenesis. Front Cell Dev Biol.

