# Supplementary material for: Identification of positive cofactor 4 as a diagnostic and prognostic biomarker associated with immune infiltration in hepatocellular carcinoma
Source: ILIVER. 2023 Sep 15;2(4):188–201. doi: 10.1016/j.iliver.2023.08.007 (PMC12212729; doi:10.1016/j.iliver.2023.08.007)
Supplement: Multimedia component 1 [file mmc1.docx]

**Fig. S1**


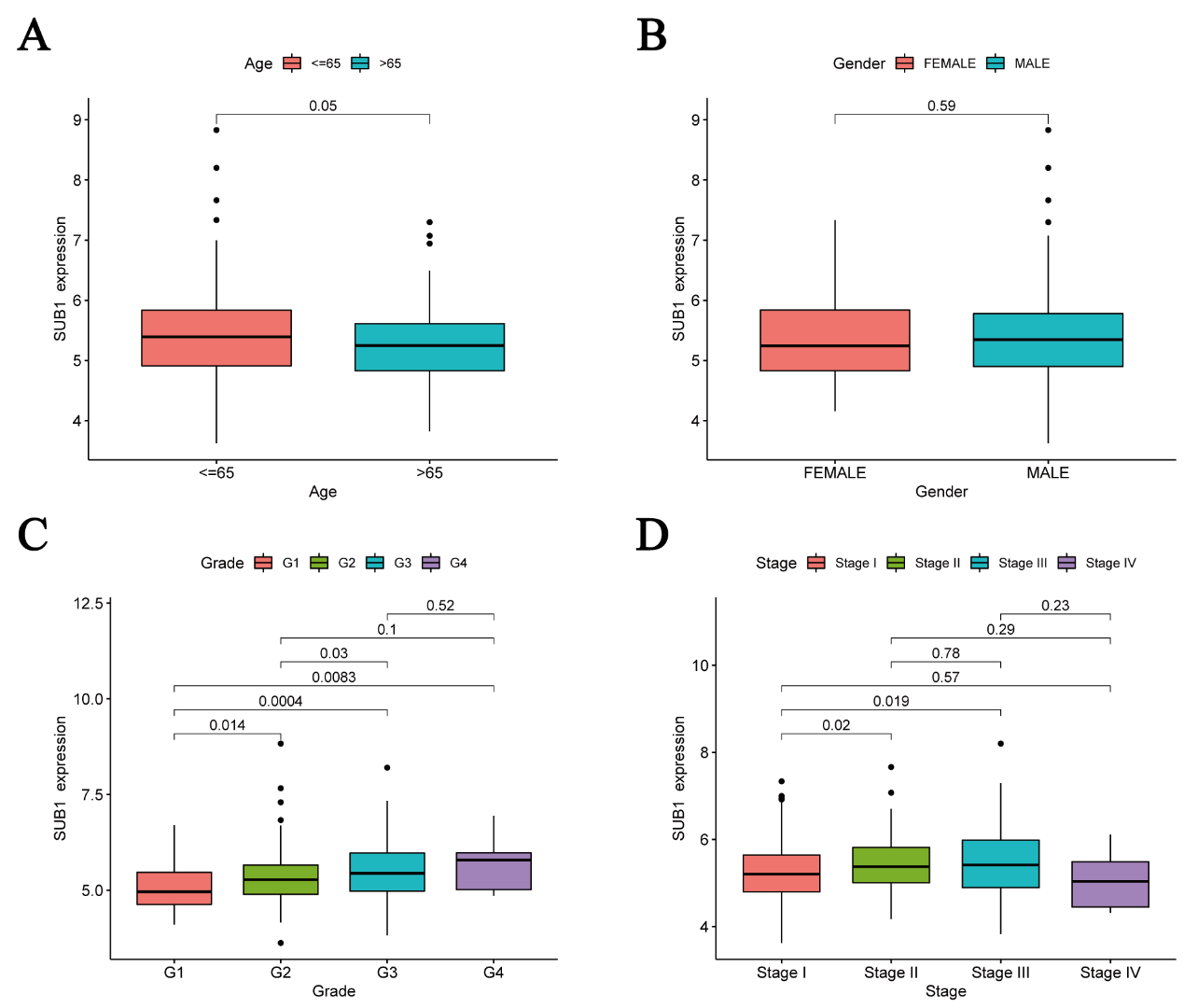


**Fig. S1** Correlation between PC4 expression level and age (A), gender (B), pathological grade (C) and clinical stage (D) of HCC patients.

**Fig. S2**


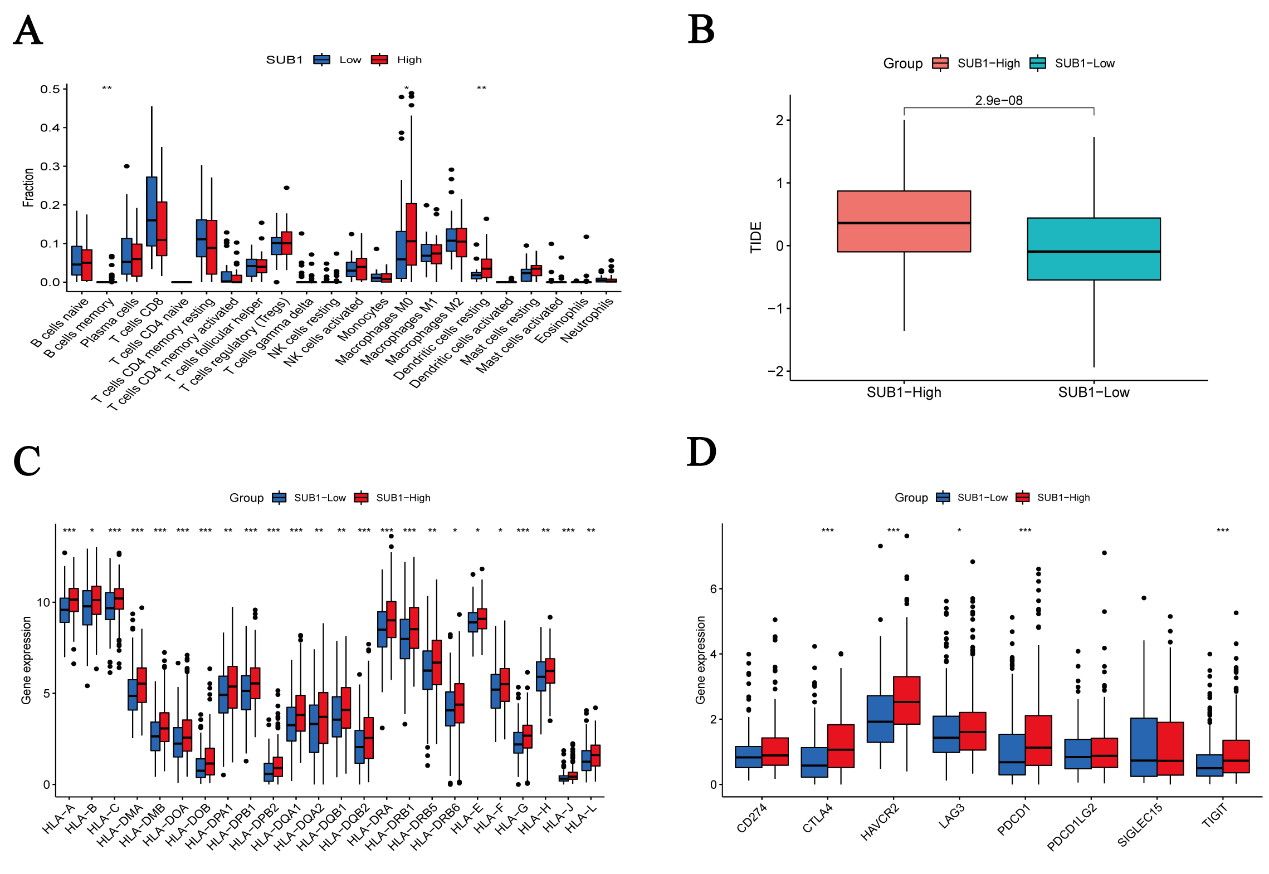


**Fig. S2** Correlation of PC4 expression level with immune cell infiltration in HCC. (A) Differences in tumor-infiltrating immune cells between the PC4-high and PC4-low groups. (B) Boxplot showing the difference in TIDE scores between the two groups. (C, D) Expression differences of 24 HLA genes (C) and 8 immune checkpoint genes (D) between the PC4-high and PC4-low groups.*p < 0.05, **p < 0.01, ***p < 0.001.

**Fig. S3**


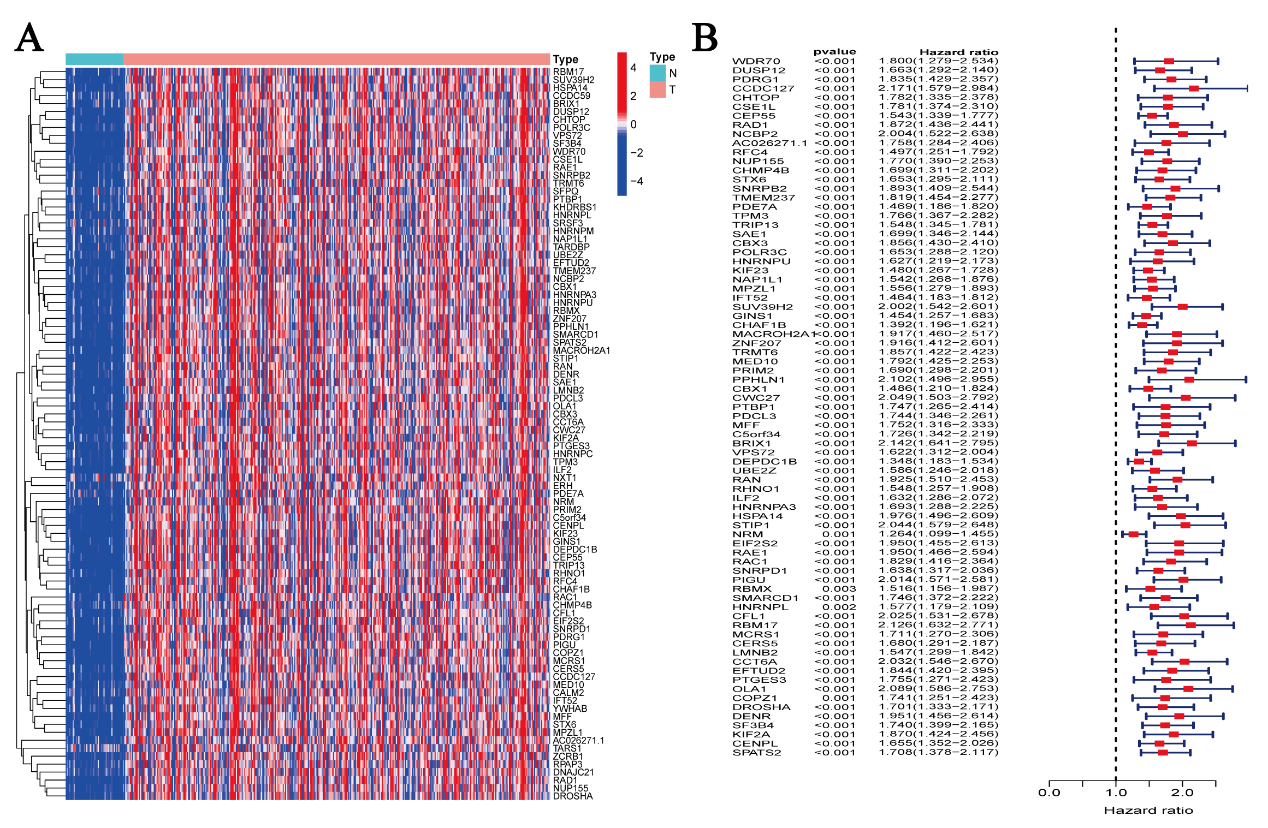


**Fig. S3** Heatmap and forest plot of PC4 co-expressed genes. (A) Heatmap showing expression of PC4 co-expressed genes in HCC and adjacent liver tissues. (B) Forest plot displaying the results of univariate COX analysis of PC4 co-expressed genes.

**Fig. S4**


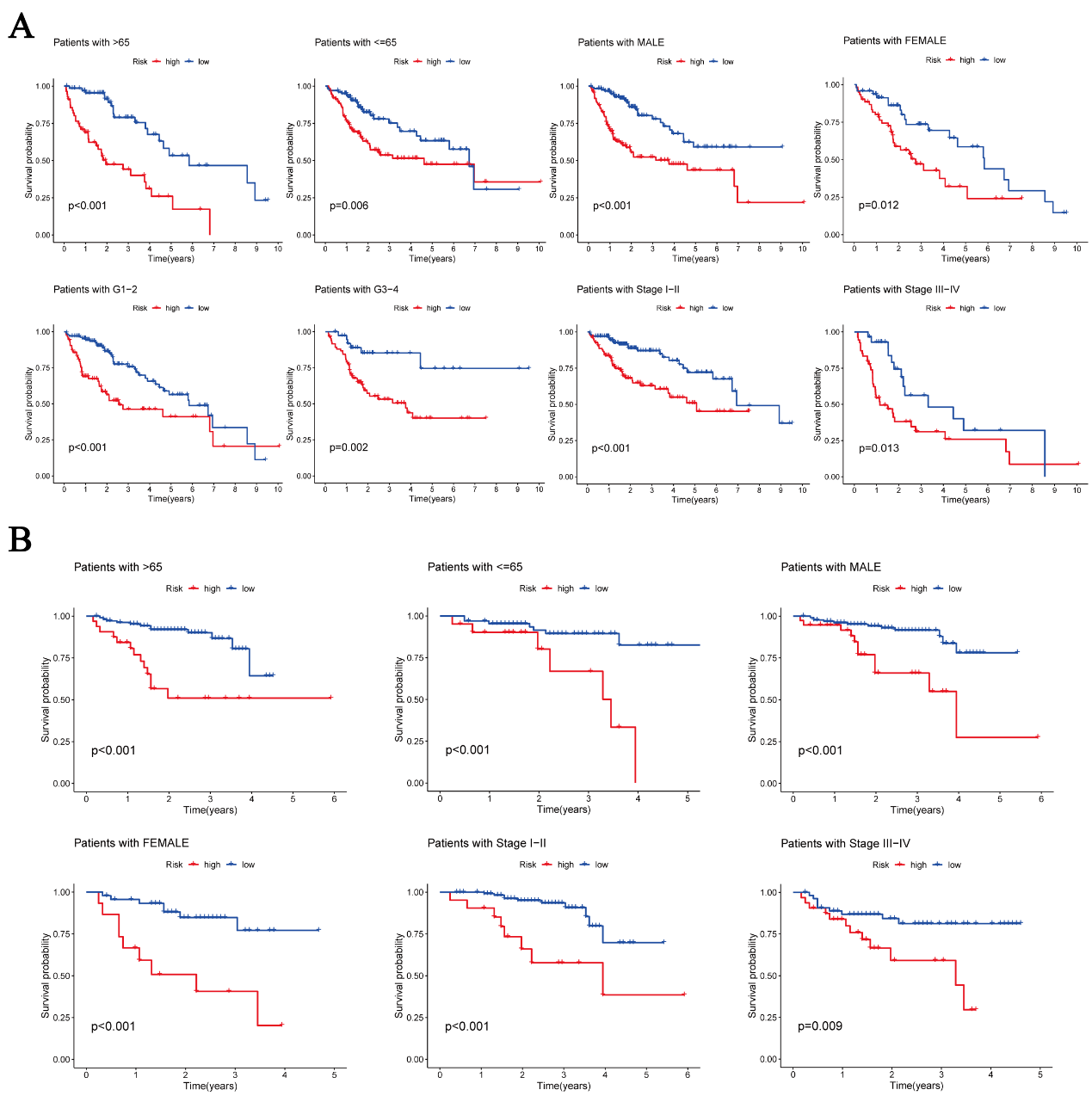


**Fig. S4** Overall survival Kaplan–Meier curves for the high-risk and low-risk groups of the training cohort (A) and test cohort (B).

**Fig. S5**


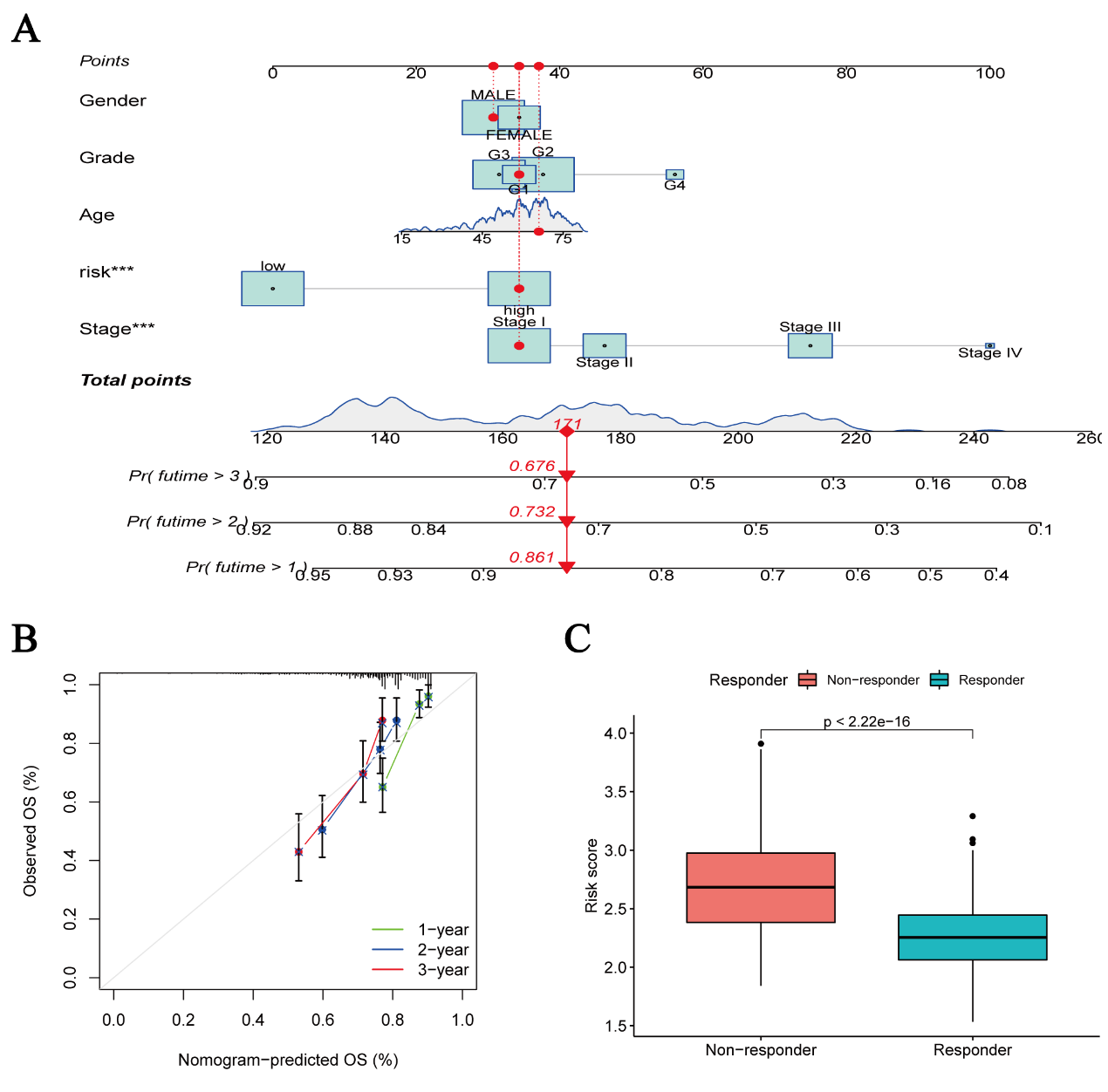


**Fig. S5** Construction of prognostic nomogram and prediction of immunotherapy response based on the training cohort (TCGA cohort). (A) Nomogram for predicting the 1-year, 2-years, and 3-years overall survival probability of HCC patients. (B) Calibration curve for evaluating the nomogram. (C) Boxplot showing the correlation between riskscore and immunotherapy response of HCC patients.

**Fig. S6**


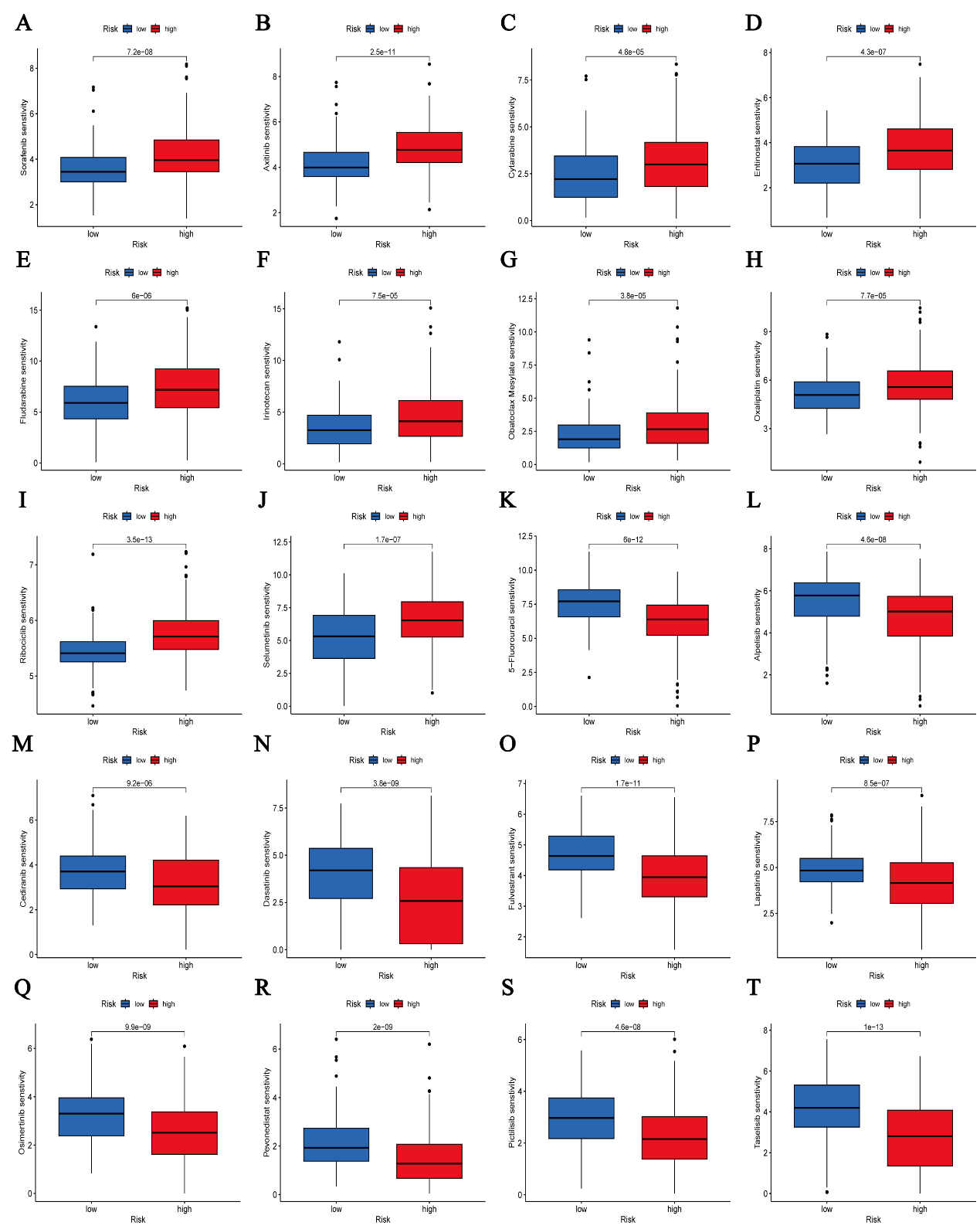


**Fig. S6** Drug sensitivity analysis between the high-risk and low-risk groups in the training cohort (TCGA cohort). (A) Sorafenib, (B) Axitinib, (C) Cytarabine, (D) Entinostat, (E) Fludarabine, (F) Irinotecan, (G) Obatoclax Mesylate, (H) Oxaliplatin, (I) Ribociclib, (J) Selumetinib, (K) 5-Fluorouracil, (L) Alpelisib, (M) Cediranib, (N) Dasatinib, (O) Fulvestrant, (P) Lapatinib, (Q) Osimertinib, (R) Pevonedistat, (S) Pictilisib, (T) Taselisib.

**Fig. S7**

**
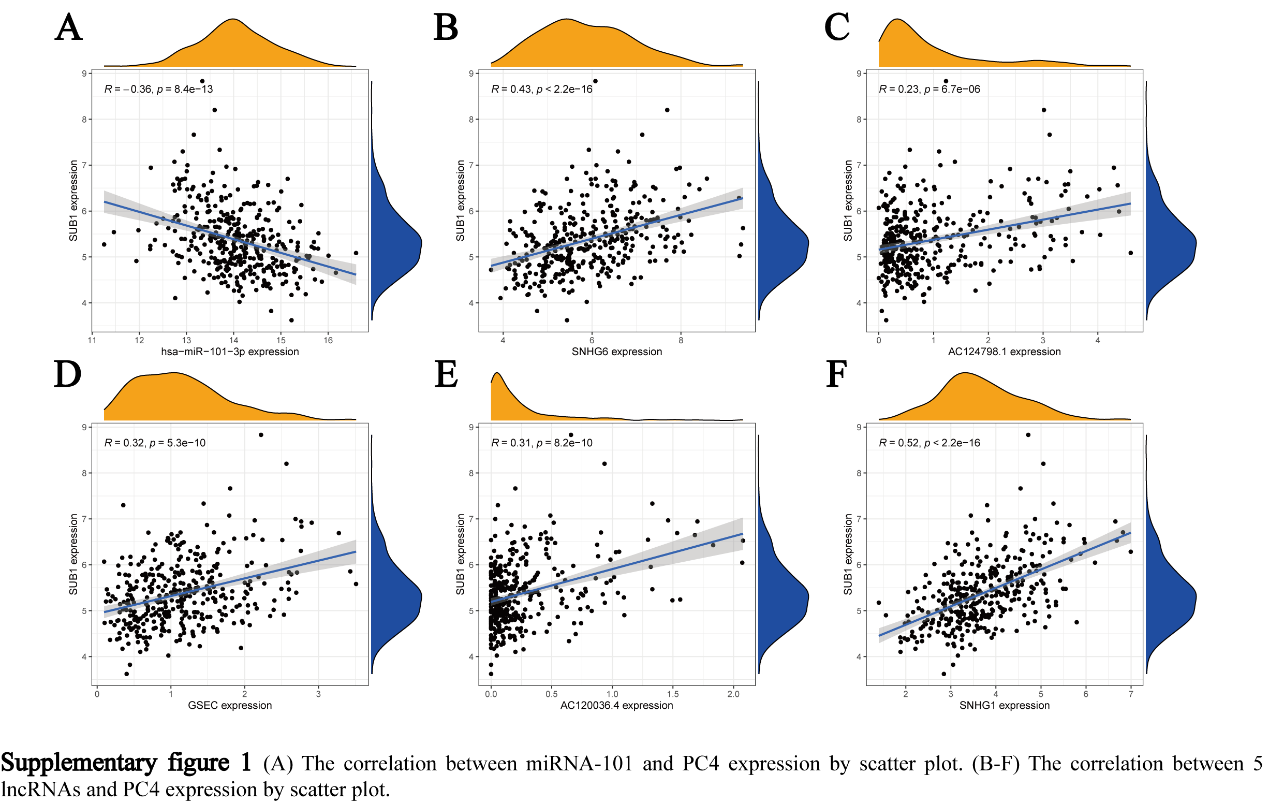
**

**Fig. S7** (A) The correlation between miRNA-101 and PC4 expression by scatter plot. (B-F) The correlation between 5 lncRNAs and PC4 expression by scatter plot.

**Fig. S8**


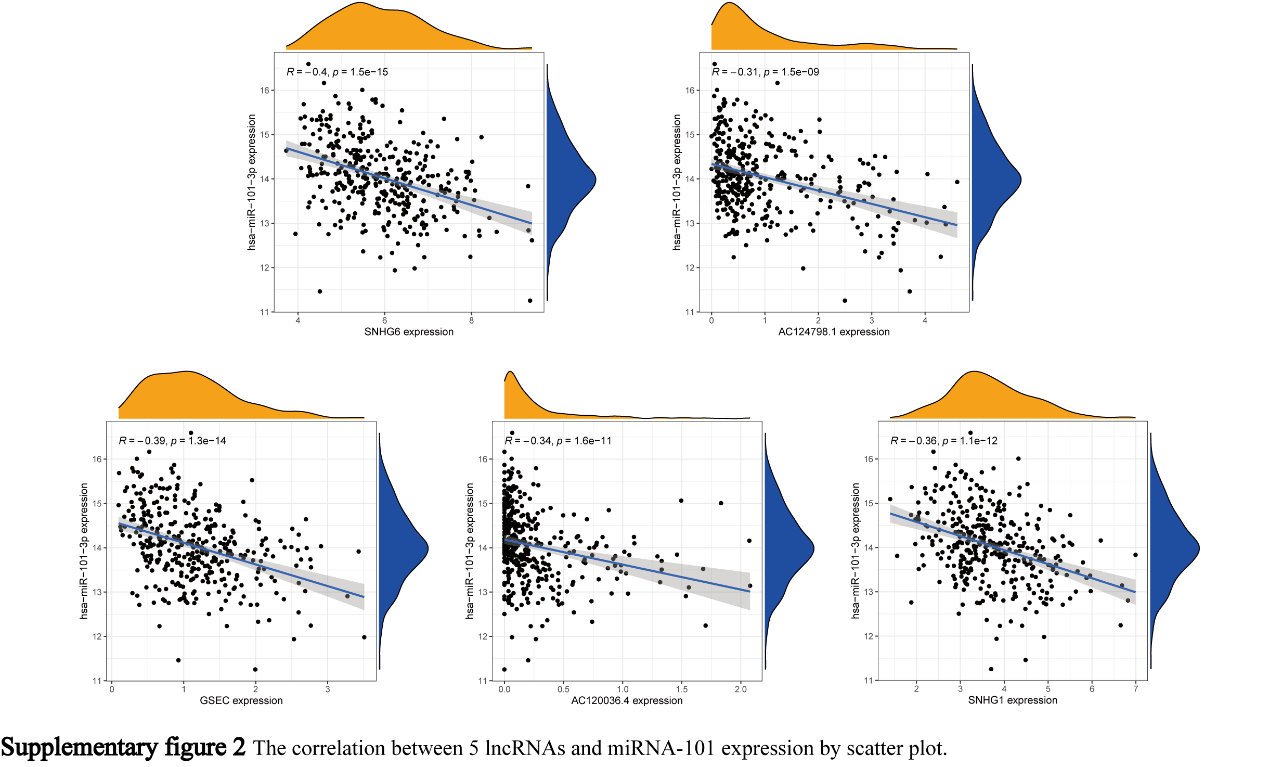


**Fig. S8** The correlation between miRNA-101 and 5 lncRNAs expression by scatter plot
