# Supplementary material for: Identification of positive cofactor 4 as a diagnostic and prognostic biomarker associated with immune infiltration in hepatocellular carcinoma
Source: ILIVER. 2023 Sep 15;2(4):188–201. doi: 10.1016/j.iliver.2023.08.007 (PMC12212729; doi:10.1016/j.iliver.2023.08.007)
Supplement: Multimedia component 4 [file mmc4.docx]

**Table S3.** The prognostic genes identified by using LASSO COX regression.

| **Gene** | **Coefficient** | **Name** |
| --- | --- | --- |
| CEP55 | 0.073654 | Centrosomal protein 55 |
| TRIP13 | 0.090714 | Thyroid hormone receptor interactor 13 |
| BRIX1 | 0.100123 | Biogenesis of ribosomes BRX1 |
| STIP1 | 0.080703 | Stress induced phosphoprotein 1 |
| PIGU | 0.191130 | Phosphatidylinositol glycan anchor biosynthesis class U |
| CFL1 | 0.037948 | Cofilin 1 |
| RBM17 | 0.016718 | RNA binding motif protein 17 |
| OLA1 | 0.017742 | Obg like ATPase 1 |
