# Supplementary material for: Identification of positive cofactor 4 as a diagnostic and prognostic biomarker associated with immune infiltration in hepatocellular carcinoma
Source: ILIVER. 2023 Sep 15;2(4):188–201. doi: 10.1016/j.iliver.2023.08.007 (PMC12212729; doi:10.1016/j.iliver.2023.08.007)
Supplement: Multimedia component 5 [file mmc5.docx]

**Tab S4.** Clinical feature of HCC patients in the training set and validation set

| **Variables** | **TCGA cohort** | | **ICGC cohort** | |
| --- | --- | --- | --- | --- |
|  | **Number** | **Percent** | **Number** | **Percent** |
| Age |  |  |  |  |
| ≤65 | 235 | 62.50% | 98 | 37.69% |
| >65 | 141 | 37.50% | 162 | 62.31% |
| Gender |  |  |  |  |
| Male | 254 | 67.55% | 192 | 73.85% |
| Female | 122 | 32.45% | 68 | 26.15% |
| Histologic grade |  |  |  |  |
| G1-2 | 235 | 62.50% | - | - |
| G3-4 | 136 | 36.17% | - | - |
| Unknown | 5 | 1.33% | - | - |
| Clinical stage |  |  |  |  |
| Stage I–II | 261 | 69.42% | 157 | 60.38% |
| Stage III–IV | 91 | 24.20% | 103 | 39.62% |
| Unknown | 24 | 6.38% | - | - |
| T stage |  |  |  |  |
| T1-2 | 279 | 74.20% | - | - |
| T3-4 | 94 | 25.00% | - | - |
| Unknown | 3 | 0.80% | - | - |
| N stage |  |  |  |  |
| N0 | 257 | 68.35% | - |  |
| N1 | 4 | 1.06% | - |  |
| Unknown | 115 | 30.59% | - |  |
| M stage |  |  |  |  |
| M0 | 272 | 72.34% | - | - |
| M1 | 4 | 1.06% | - | - |
| Unknown | 100 | 26.60% | - | - |
| Survival status |  |  |  |  |
| Alive | 244 | 64.89% | 214 | 82.31% |
| Dead | 132 | 35.11% | 46 | 17.69% |
